# Do Times until Treatment for Foreign Body Aspiration Relate to Complications?

**DOI:** 10.1155/2016/2831614

**Published:** 2016-08-17

**Authors:** Walailak Tatsanakanjanakorn, Surapol Suetrong

**Affiliations:** Department of Otorhinolaryngology, Faculty of Medicine, Khon Kaen University, Khon Kaen 40002, Thailand

## Abstract

*Introduction*. Foreign body aspiration is an emergency condition and may be fatal. Delayed diagnosis and treatment may be associated with complications. This study evaluated the association between time until treatment and complications due to foreign body aspiration.* Methods*. This study was a retrospective study conducted at Khon Kaen University Hospital, Thailand. We enrolled patients diagnosed with foreign body aspiration with evidence of foreign body detected using direct laryngobronchoscopy at any area from the larynx to the bronchus. Descriptive statistics were used to analyze the association of times of treatment with complications of foreign body aspiration.* Results*. During the study period, there were 43 patients that met the study criteria. The most common age group was 0–2 years. Plant seeds were the most common foreign bodies (41.9%), and the right main bronchus was the most common site (16 patients, 37.2%). There were 30 patients (69.8%) that experienced complications from foreign body aspiration. Pneumonia was the most common complication (14 patients, 32.6%). The retention time was not significantly associated with the presence of complications (*p* value: 0.366). Two patients (4.7%) died due to complete airway obstruction and prolonged hypoxia.* Conclusion*. Times until treatment were not significantly associated with complications from foreign body aspiration.

## 1. Introduction 

Foreign body aspiration (FBA) is an emergency condition and may be fatal [1]. This condition is more common in children. It has been estimated that 500 children die from foreign body aspiration each year in the USA [2]. The overall mortality rate of foreign body aspiration is approximately 5–7% [3]. FBA with complete airway obstruction is responsible for majority of the mortality cases immediately after the injuries [1, 4–7]. Although FBA with incomplete airway obstruction is not a cause of instant death, it can result in significant morbidities, complications, and delayed deaths [8]. The complications may be associated with many risk factors: foreign body (FB) sites, FB characteristics (size, shape, surface, edge, consistency, and types: organic, inorganic), child's characteristics, socioeconomic status, delayed diagnosis, and delayed management [9, 10]. Delayed diagnosis and management are the main causes of serious complications regarding foreign body aspiration, particularly in children [11, 12]. The main reason for misdiagnosis or delayed diagnosis is a lack of specific signs or symptoms. About 15–20% of patients may not have any signs or symptoms of foreign body aspiration [13], while others may have symptoms that mimic those of pneumonia or asthma [14–18].

The complication rate of foreign body aspiration in children varies among studies from 14.6 to 27.8% [19, 20]. Pneumonia and respiratory distress were common complications. There are limited data on risk factors associated with complications of foreign body aspiration in children. A report from China [20] showed that the type of foreign body and symptoms were associated with respiratory complications. This study aimed to evaluate whether or not delayed treatment may be a risk factor for complications of foreign body aspiration.

## 2. Method

This study was a retrospective study conducted at Khon Kaen University Hospital, Thailand. The study period was between January 1997 and December 2006. We enrolled patients diagnosed with foreign body aspiration with evidence of foreign bodies detected using direct laryngobronchoscopy at any area from the larynx to the bronchus. Patients were excluded if medical records were incomplete or if the foreign body was found elsewhere such as the nose, nasopharynx, or esophagus.

Medical records of all eligible patients were reviewed. Demographic data, presenting symptoms, clinical findings, types of foreign bodies, and complications stemming from foreign body aspiration were recorded. Descriptive statistics were used to analyze clinical features and reported as percentage. The association between times until treatment and presence of complications was tested by Fisher's exact test using SPSS Statistics 17.0 software. *p* values less than 0.05 were considered as statistically significant.

## 3. Results

During the study period, there were 103 patients diagnosed with foreign body aspiration. Among the 60 patients who were excluded, 28 were excluded due to nonlaryngotracheopulmonary FB, 17 were excluded due to incomplete medical records, and 15 were excluded due to the fact that no FB was identified. In total, 43 patients met the study criteria.

Clinical features are listed in Table 1. The most common age group was 0–2 years. Coughing (72.1%) and choking (67.4%) were the most two common presenting symptoms, while decreased breath sound was the most common physical sign (62.8%). Hyperaeration was the most common CXR finding (39.5%). Normal CXR was found in 8 patients (18.6%).

According to bronchoscopic findings (Table 2), plant seeds were the most common foreign bodies (41.9%), and the right main bronchus was the most common site (16 patients, 37.2%).

There were 30 patients (69.8%) who experienced complications from foreign body aspiration (Table 3). Pneumonia was the most common complication (14 patients, 32.6%).

The retention times ranged from less than 24 hours to more than 15 days. Over a half of patients (62.5%) had a retention time of more than 24 hours (Table 4).

The patients with retention time of more than 24 hours had over 2 times more complications than those with retention time of less than 24 hours (15 : 6; Table 4). But there was no significant association between times until treatment and presence of complications (*p* value of 0.366 by Fisher's exact test).

Two patients (4.7%) died from complete airway obstruction and prolonged hypoxia. One patient aspirated chicken bone and had immediate respiratory failure for 20 minutes with cardiac arrest at the community hospital, at which bronchoscopy was not available. The other patient suffered from foreign body aspiration at the right main bronchus with failure to remove the foreign body by bronchoscopy at the local general hospital. During the transfer, the patient developed sudden severe airway obstruction for several hours prior to cardiac arrest.

## 4. Discussion 

This was a retrospective study conducted at the referral center in northeast Thailand using data collected over a 10-year period. Clinical features of patients with foreign body aspiration were comparable to those described in previous studies [11, 12]. Most patients were young children with nonspecific symptoms/signs/and CXR findings [21]. A history of choking, poor air entry on examination, localized wheezing, and localized atelectasis may suggest foreign body aspiration. Note that normal CXR was found in 18.6% of patients (Table 1). CT may be helpful in the diagnosis of foreign body aspiration in cases of normal CXR [20].

The mortality rate in this study was 4.7%, which was higher than those in previous reports [22, 23]. Both of these previous reports were from India. The first one looked at 37 children who suffered from foreign body aspiration with no mortality, while the second report involved 140 children with a mortality rate of 0.7%. Our mortality rate was high, because we included the fatality cases of primary and secondary hospital setting. These 2 patients died before reaching our facilities, the death of whom might be explained by lacking of proper medical instruments, inadequate experiences of physicians managing this problem, or severity (complete or nearly complete obstruction) of disease itself. However, our mortality rate was still in the global range (5–7%) reported by Foltran et al. [3]. Bamber et al. [1] reported that 60% of the mortality cases died immediately after the event with no time for resuscitation, as did one of the cases we examined. Complete or near-complete airway obstruction was the primary mechanism by which this occurred. The remaining (40%) had a prolonged survival time after treatment before death which is caused by hypoxic ischemic injury [1] just as another case of our study. Other mechanisms [24] included reflex cardiopulmonary arrest triggered by vagal stimulation and the esophageal foreign body compressing the posterior membranous trachea, causing airway obstruction. Delayed treatment due to a lack of recognition may be another factor associated with death or complications [3]. Our data (Table 4) showed no significant association between times until treatment and numbers of patients with complications from foreign body aspiration. However, complication rate was over 2 times higher in prolonged retention time of more than 24 hours. This was comparable to the study of Shlizerman et al. [11] in which rate of complications increased twofold for patients with delayed treatment time more than 2 days. But this increased complication was not statistically significant.

There are some limitations in this study. First, the sample size was quite small, despite the fact that this hospital-based study was performed at the university tertiary care hospital. This may explain the low prevalence of foreign body aspiration. Also, some data were missing due to the data collection being retrospective. Further prospective studies are required to evaluate the association between times of treatment and complications of foreign body aspiration. This study evaluated the association of times until treatment and overall complications and was not specific to individual complications, as shown in Table 3.

## 5. Conclusion

Time until treatment was not significantly associated with complications from foreign body aspiration.

## Figures and Tables

**Table 1:** Clinical characteristics of patients with foreign body aspiration (*n* = 43).

Characteristics	Frequency	Percentage
Age, years		
0–2	25	58.1
3–7	5	11.6
8–12	6	14.0
>12	7	16.3

Presenting symptoms^*∗*^		
Cough	31	72.1
Choking	29	67.4
Fever	7	16.3
Hoarseness	3	7.0
Others, that is, vomiting and sore throat	3	7.0

Physical signs^*∗*^		
Decreased breath sound	27	62.8
Rhonchi	9	20.9
Wheezing	9	20.9
Stridor	4	9.3
Cyanosis	1	2.3
Chest retraction	5	11.6
Normal physical exam	7	16.3

Chest X-ray		
Hyperaeration	17	39.5
Atelectasis	7	16.3
Pulmonary infiltration	2	4.7
Radioopacity	4	9.3
Normal	8	18.6
No data	5	11.6

Note: *∗* indicates that one patient may have more than one symptom/sign.

**Table 2:** Types and sites of foreign body aspiration in children (*n* = 43).

	Frequency	Percentage
Types of foreign body		
Plant seeds	18	41.9
Peanut seed	9	20.9
Fish bone	3	7.0
Chicken bone	2	4.7
Tooth	3	7.0
Pork belly	2	4.7
Coin	1	2.3
Others^*∗*^	5	11.6

Sites of foreign body		
Right main bronchus	16	37.2
Left main bronchus	12	27.9
Right second bronchus	3	7.0
Left second bronchus	3	7.0
Glottis	6	14.0
Trachea	1	2.3
Multiple sites	2	4.7

Note: *∗* included pen caps, a candy, a whistle, marbles, and a T-tube.

**Table 3:** Complications of foreign body aspiration in children (*n* = 43).

Complications	Frequency	Percentage
Pneumonia	14	32.6
Hypoxia	9	20.9
Ventilatory support	6	14.0
Laryngeal stenosis	1	2.3

**Table 4:** Times until treatment and presence of complications (*n* = 40; no data = 3).

Times until treatment	With complications	Without complications	Total
<24 hours	6	9	15
1-2 days	5	3	8
3–7 days	3	5	8
8–14 days	2	0	2
>15 days	5	2	7

Total	21	19	40
